# Intrauterine Bleomycin Administration for Fetal Lymphatic Malformation: A Novel Therapeutic Approach—Case Report and Literature Review

**DOI:** 10.3390/jcm15124467

**Published:** 2026-06-09

**Authors:** Marcelina Sztyler-Krakowska, Aleksandra Sliwka, Emilia Piotrkowicz, Remigiusz Krysiak, Jarosław Żyłkowski, Bartosz Godek, Przemysław Kosinski

**Affiliations:** 1 Fetal Therapy and Perinatology Students’ Research Group, Medical University of Warsaw, 02-097 Warsaw, Poland; marcelina.sztyler@gmail.com (M.S.-K.); aleksandra.sliwka@wum.edu.pl (A.S.); 2 Department of Obstetrics, Perinatology, Gynecology and Reproductive Medicine, Medical University of Warsaw, 02-097 Warsaw, Poland; 3 Second Department of Radiology, Medical University of Warsaw, 02-097 Warsaw, Poland

**Keywords:** lymphatic malformations, fetal therapy, perinatal lymphangiomas

## Abstract

Perinatal lymphangiomas are rare benign congenital malformations of the lymphatic system, whose potential for rapid growth and local invasiveness may pose significant risks to fetal well-being. This report presents a case of a large fetal lymphangioma diagnosed prenatally during a second-trimester ultrasound examination. The lesion was initially asymptomatic but subsequently progressed, resulting in ascites and pericardial effusion. In light of progressive fetal deterioration, prenatal sclerotherapy was performed using ultrasound-guided transabdominal administration of bleomycin. Despite the technical success of this procedure, the neonate developed severe cardiorespiratory failure and died shortly after delivery. This case highlights both the potential benefits and limitations of prenatal intervention in severe lymphatic malformations. This study also includes a concise review of current perinatal and postnatal management strategies. Despite advances in prenatal imaging and therapy, further studies are needed to optimize treatment and improve neonatal outcomes.

## 1. Introduction

Perinatal lymphatic malformation is a congenital disorder characterized by impaired lymphatic drainage, frequently manifesting as a benign mass. The etiology of this condition is heterogeneous, involving either impaired lymphatic–venous connections or abnormal lymphatic vessel formation [1,2]. During embryogenesis, a lymphangioma may arise due to sequestration of the primitive lymphatic sac from the rest of the lymphatic system, disturbances in lymphatic or venous drainage, abnormal proliferation of lymphatic endothelial cells or obstruction within the lymphatic vessels [3]. Cystic hygroma can be detected prenatally in the first trimester of pregnancy and, in approximately 50 percent cases, is associated with chromosomal abnormalities, such as Down syndrome, Turner syndrome, and Noonan syndrome [4]. Lymphangiomas are highly variable in size, ranging from a few millimeters to over 10 cm, and may present as either unilocular or multilocular lesions [3]. Perinatal lymphatic malformation is considered rare, with an estimated incidence of approximately 1 per 6000 live births [1]. This condition usually develops during the second and third trimesters of gestation and may present either during pregnancy or shortly after delivery [3,5]. The frequency of spontaneous intrauterine regression of fetal lymphangiomas has not been clearly established. However, available evidence suggests that such outcomes are exceptionally rare [6].

Although lymphatic malformations are most commonly located in the cervical region, they can also occur in other anatomical sites, including the axillary region, limbs, and abdominal wall [5]. The differential diagnosis includes a hemangioma, cervical teratoma, congenital goiter, and thyroid cyst [2].

While perinatal lymphatic malformations are typically considered benign, their potential for rapid growth and tendency to invade surrounding structures emphasize the significance of early prenatal detection as a vital aspect of management [2,5]. Prognosis of this condition is highly variable and depends on factors such as the lesion’s location and extent, the presence of an abnormal karyotype, and other associated abnormalities [1]. Lesions situated in the cervical region can lead to fetal upper airway obstruction, requiring intrapartum airway interventions [2]. The timing of diagnosis is significant, as lesions that develop earlier in gestation are frequently linked to a more severe clinical course [1]. Early identification facilitates comprehensive parental counseling and enables informed planning of the clinical strategy, including the option of pregnancy termination, optimization of delivery planning, and preparation for postnatal care [5].

Diagnosis of lymphatic malformations can be made using various imaging techniques including prenatal ultrasound, magnetic resonance imaging (MRI), and postnatal computed tomography (CT). Definitive pathological confirmation can be obtained postoperatively or on autopsy [3]. Ultrasound serves as the primary tool for prenatal detection, with lymphatic malformations typically presenting as multicystic, multiseptated structures of variable shape and size [2]. Characteristic ultrasound findings include a septated cystic mass without detectable blood flow on color Doppler imaging [1]. Certain locations of lymphatic malformations, such as the anterior neck region, might require fetal MRI to facilitate intrapartum management [7]. Notably, the earliest—or only—prenatal manifestation of a lymphatic malformation may be pericardial effusion, defined as an abnormal accumulation of fluid within the pericardial sac exceeding physiological levels. This condition most commonly results from impaired lymphatic drainage or increased fluid production [8,9].

In particular cases, lymphatic malformations may be associated with other fetal anomalies, including aneuploidy, and can lead to subsequent pregnancy complications, such as hydrops fetalis and fetal growth restriction. The genetic background of these lesions varies according to their type and time of detection. Cystic hygromas detected in the first or early second trimester are frequently linked to chromosomal abnormalities, including trisomy 13, 18, and 21, whereas isolated anterolateral lymphangiomas identified after 30 weeks of gestation are typically not related to chromosomal defects and generally carry a more favorable prognosis. This heterogeneity contributes to a more complex clinical presentation, requiring a multidisciplinary approach to management [1,2,10].

## 2. Case Presentation

A 29-year-old patient, gravida 2, para 1, was referred to the Department of Obstetrics, Perinatology, Gynecology, and Reproductive Medicine at the Medical University of Warsaw for a second opinion and counseling following abnormal findings on a routine second-trimester ultrasound scan. The course of the pregnancy up to that point had been uneventful, and the mother had no underlying chronic diseases.

A detailed ultrasonographic assessment performed transabdominally at 22 + 6 weeks of gestation revealed a multilocular, mixed cystic lesion extending from the level of the left fetal iliac crest to the left knee, with involvement of the lower abdominal cavity. This lesion measured 958 mm in diameter, demonstrated weak internal vascularity on Doppler examination, and was interpreted as a massive fetal lymphatic malformation. Fetal biometry corresponded to gestational age, fetal movements were normal, and the amniotic fluid index (AFI) remained within normal limits. No additional structural anomalies were identified during the ultrasonographic examination, and the assessed fetal karyotype was normal.

**Figure 1:**
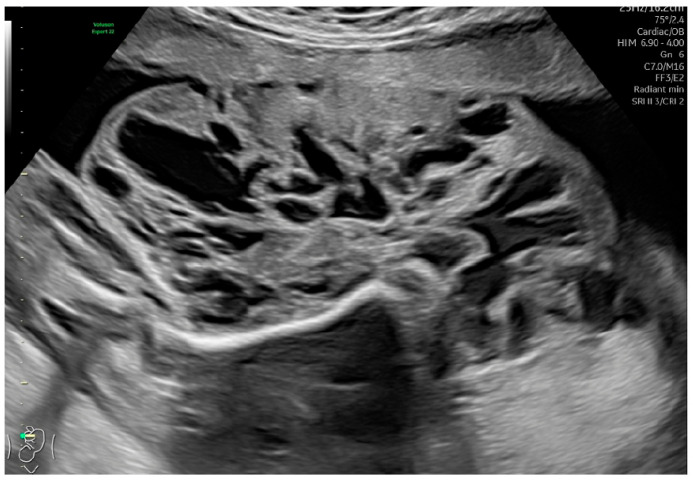
Fetal femur and massive lymphatic malformation of the lower limb. Complete visualization of the malformation in a single image was not technically feasible due to the extensive size of the lesion.

A fetal MRI was subsequently performed to better define the extent and characteristics of the malformation. The examination confirmed the presence of an isolated lymphatic mass extending from the left iliac crest to the left knee of the fetus. Ongoing monitoring was planned as part of the continued care strategy, including serial fetal ultrasonography, fetal MRI, and multidisciplinary assessments. In the following weeks, the fetus presented ultrasonographic signs of distress, including progressive severe ascites and pericardial effusion, indicating an increased risk to fetal well-being and survival (Figure 1).

The high risk of intrauterine fetal demise and the remarkable size of the lymphatic mass led to a decision to perform prenatal sclerotherapy at 28 weeks of gestation. Bleomycin was selected for the procedure, because of its well-characterized mechanism of action and extensive postnatal safety and efficacy data. While bleomycin has not been previously reported in the prenatal setting, its established effectiveness in postnatal management supported its use in this case. In our center all neonates diagnosed with lymphatic malformations are treated postnatally with bleomycin injections with good outcomes. In this context, the application of bleomycin can be interpreted as a translational approach, extending a well-established postnatal therapy to fetal life in a situation of severe disease progression and limited therapeutic alternatives. To prioritize fetal and maternal safety, the minimal effective dose reported in recent studies was selected. The dosage was calculated based on the fetal weight, with a minimum dose of 1 unit of bleomycin per 1 g of neonatal weight. Hence, we administered 1500 units based on the estimated fetal weight of 1498 g at the time of treatment.

The procedure was performed using a 22G needle introduced transabdominally under continuous ultrasound guidance. Upon verifying proper needle placement, a sclerosing agent (bleomycin) was administered precisely within the malformation, minimizing the risk of systemic exposure to the fetus, placenta, or maternal bloodstream. The prenatal procedure we performed was similar to the postnatal management of lymphatic malformations, differing only in the timing of the sclerosing agent’s administration.

Post-treatment ultrasonography and cardiotocography were performed to confirm fetal well-being. Due to the potential side effects of bleomycin, maternal renal and hepatic functions were evaluated the day after the procedure and prior to hospital discharge. Anticipated outpatient follow-up visits were scheduled to monitor the intervention’s effectiveness in reducing the sizes of the malformations. Importantly, prenatal administration of bleomycin was not associated with any maternal adverse events. No obstetric complications occurred, and treatment did not require discontinuation due to toxicity or intolerance. The maternal safety profile observed in this case is consistent with the established pharmacological data and known safety characteristics of bleomycin. Our team of pediatric radiologists planned to continue the treatment with bleomycin in postnatal life.

The rationale for the use of bleomycin in the prenatal setting is based on its well-established mechanism of action as a sclerosing agent, inducing endothelial damage, inflammation, and subsequent fibrosis within cystic structures. This mechanism is directly applicable to lymphatic malformations, which consist of abnormal lymphatic channels and cystic spaces.

In postnatal practice, bleomycin is widely used with a favorable safety and efficacy profile, particularly in large or infiltrative lesions. Importantly, its local administration allows for targeted treatment, minimizing systemic exposure. In our case, the drug was administered under continuous ultrasound guidance directly into the lesion, with the aim of reducing the risk of fetal and maternal systemic effects.

Although data on prenatal use are lacking, the decision to extrapolate postnatal experience to the fetal setting was supported by the life-threatening progression of the lesion and the absence of established prenatal treatment protocols. Similar translational approaches have been described for other prenatal interventions in lymphatic malformations, such as OK-432 or maternal sirolimus therapy.

Nevertheless, it must be emphasized that this approach remains experimental, and further studies are required to evaluate its safety, optimal dosing, and long-term outcomes.

During a routine third-trimester ultrasound examination, placentomegaly with features of placental edema was identified. Doppler assessment revealed an increased uterine artery pulsatility index (UtA PI) exceeding the 95th percentile for gestational age, indicating impaired uteroplacental perfusion.

At 31 weeks of gestation, the patient experienced significant uterine contractions and elevated blood pressure. Laboratory tests revealed the following: C-reactive protein (CRP): 3.89 mg/dL, procalcitonin (PCT): 1.060 ng/mL (normal <0.05 ng/mL), white blood cells (WBC): 15.64 × 10^3^/µL (normal 4.00–10.00 × 10^3^/µL), ALT: 540 U/L (normal 7–56 U/L), AST: 558 U/L (normal 5–40 U/L), uric acid: 9.2 mg/dL (normal 2.4–5.7 mg/dL), creatinine: 1.23 mg/dL (normal 0.50–0.90 mg/dL), urinary leukocytes: 18 cells/µL, and numerous bacteria in the urine. Urea levels and platelet counts were within normal limits, and no proteinuria was detected. Based on laboratory and imaging findings (including fetal edema, placental edema, placentomegaly, and signs of preeclampsia), the patient was diagnosed with Ballantyne syndrome [11].

Ballantyne syndrome (also known as the mirror syndrome) is a rare condition characterized by the coexistence of fetal hydrops- defined as abnormal fluid accumulation in at least two fetal compartments- and maternal manifestations that “mirror” the fetal edema. Maternal diagnostic criteria for Ballantyne syndrome are less well defined and remain heterogeneous across published reports [11]. Some authors describe the diagnosis in the context of rapid maternal weight gain and generalized edema, whereas others require a preeclampsia-like clinical presentation, including hypertension and proteinuria [12,13,14]. Although standardized maternal criteria are lacking, mirror syndrome should be considered in any pregnancy complicated by fetal hydrops in the presence of associated maternal clinical or laboratory abnormalities. The most common diagnostic criteria, according to the systematic review by Biswas et al., are presented in Table 1.

**Figure 2:**
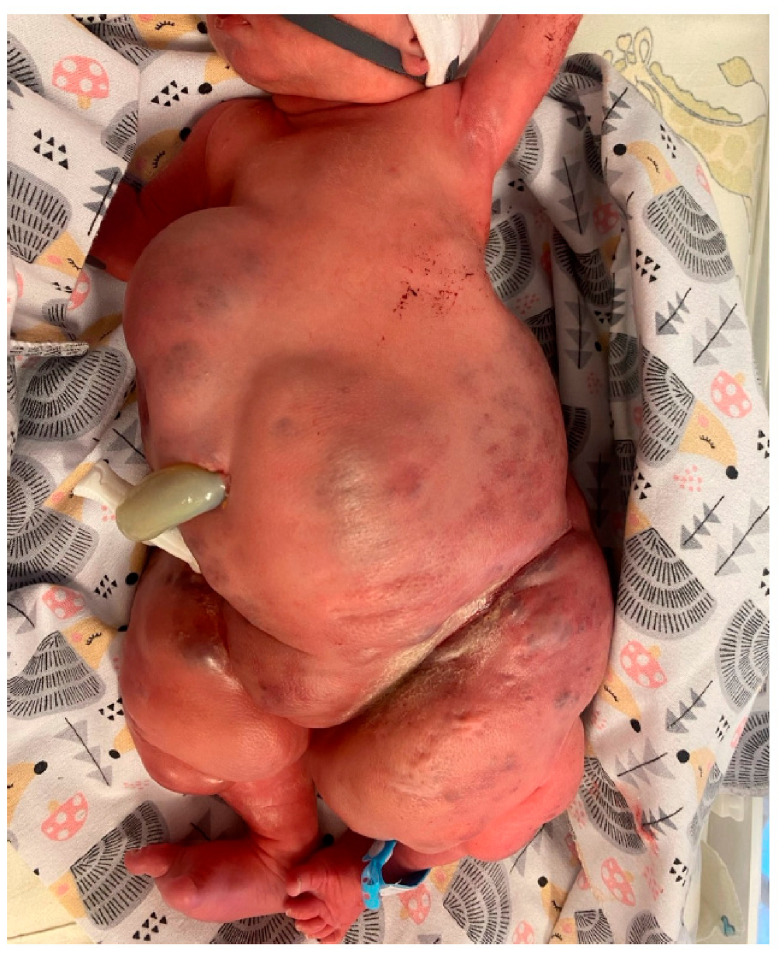
Massive lymphatic malformation in the neonate.

The presence of Mirror Syndrome, associated with hypertension, elevated creatinine levels, and increased uterine artery pulsatility index (UtA PI), prompted the decision to end the pregnancy via cesarean section at 30 + 4 weeks of gestation. A premature male neonate was born with moderate asphyxia and cardio-respiratory failure, requiring non-invasive respiratory support. Umbilical cord blood gas analysis showed a pH of 7.32 and a base excess (BE) of −3.1. The newborn weighed 3880 g (100th percentile), reflecting massive lymphatic malformations consistent with the prenatal imaging findings and extensive generalized edema (Figure 2). Neonatal examination immediately after birth revealed a head circumference of 30 cm (91st percentile) and a body length of 45 cm (98th percentile). The lymphatic malformation (extending from the level of fetal head to the left knee, with massive involvement of the lower abdominal cavity) constituted approximately two-thirds of the neonate’s total body volume. The mass continued to enlarge over subsequent days. Surgical intervention was deemed unfeasible due to the critical condition of the neonate. The patient was hemodynamically unstable, requiring aggressive fluid resuscitation, intravenous catecholamines, and steroid therapy. The neonate received continuous antibiotic therapy and opioid analgesia throughout the stay in the neonatal intensive care unit. Performed echocardiography revealed signs of pulmonary hypertension. In the following days, hematological abnormalities were detected, necessitating transfusions of blood products. Despite all undertaken interventions, the treatment was unsuccessful, and the patient died on day 4 due to heart failure.

Ultimately, our therapy might not have altered the outcome due to the extensive and multifocal nature of the lymphatic malformation, severity of fetal compromise (including ascites, pericardial effusion, and hydrothorax), late gestational age at intervention, and short time before delivery. This case underscores the challenges of managing significant prenatal lymphatic malformations and highlights the potential application of sclerotherapy in altering the course of fetal development. Our study serves as a foundational step for future research, demonstrating that prenatal administration of bleomycin is feasible and not associated with significant maternal adverse effects. Further analysis is warranted to refine prenatal therapeutic approaches and timing to improve neonatal outcomes in similar cases.

## 3. Perinatal Lymphatic Malformations Treatment: A Literature Review

### 3.1. Materials and Methods

This review is a structured narrative review with a systematic search synthesizing current knowledge on treatment strategies for perinatal lymphatic malformations. Although a systematic literature search was conducted, the review does not strictly follow PRISMA guidelines, as the aim was narrative synthesis and interpretation of findings.

The aim of this study was to systematically summarize the current evidence on prenatal management strategies for fetal lymphatic malformations. The research question guiding this review was: ”What are the current approaches to the prenatal management of fetal lymphatic malformations?” The review question was structured according to the PICO framework:

Population (P): fetuses diagnosed with lymphatic malformations.

Intervention (I): prenatal management strategies, including sclerotherapy, pharmacologic treatment, and drainage procedures.

Comparison (C): alternative postnatal approaches or expectant management, when available.

Outcome (O): fetal and neonatal outcomes, including survival, complication rates, and reduction in lesion size.

A systematic literature search was conducted between February 2025 and March 2025 across multiple databases, including PubMed and Scopus as the primary sources, while Google Scholar was used only as a supplementary search tool. The last literature search was performed on 21 March 2025. The search strategy employed specific keywords and Boolean operators related to prenatal lymphatic malformations and their management. The following terms were used: (“Prenatal Diagnosis” OR prenatal OR fetal OR antenatal OR “in utero”) AND (“Lymphangioma” OR “Lymphatic Vessel Malformations” OR lymphangioma* OR “lymphatic malformation*” OR “cystic hygroma”) AND (treatment OR management OR therapy). The search was performed simultaneously by two authors (A.Ś. and M.S.), and was modified according to the technical requirements of each database. Full-text analysis and data extraction were performed in a standardized way by two authors (A.Ś. and M.S.). Any potential disagreements within a pair of co-authors were resolved by consultation with a third author (E.P.).

This review includes studies published within the last decade to ensure inclusion of the most current evidence. Studies were selected based on predefined inclusion criteria:

1. Case reports and case series focused on lymphatic malformations in neonates and infants.
2. Research articles discussing diagnostic techniques, management strategies, and outcomes for patients with lymphatic malformations.

Exclusion criteria included:

1. Articles in a language other than English.
2. Publications lacking original data.
3. Research focusing on adult patients or non-lymphatic malformations.
4. Papers focusing exclusively on diagnostic aspects without reporting management or outcomes.

Data from the selected studies were extracted systematically, focusing on key parameters such as patient demographics, clinical presentation, diagnostic methods, treatment approaches, and outcomes. The synthesis of these findings allows for a nuanced understanding of lymphatic malformations and their management.

**Figure 3:**
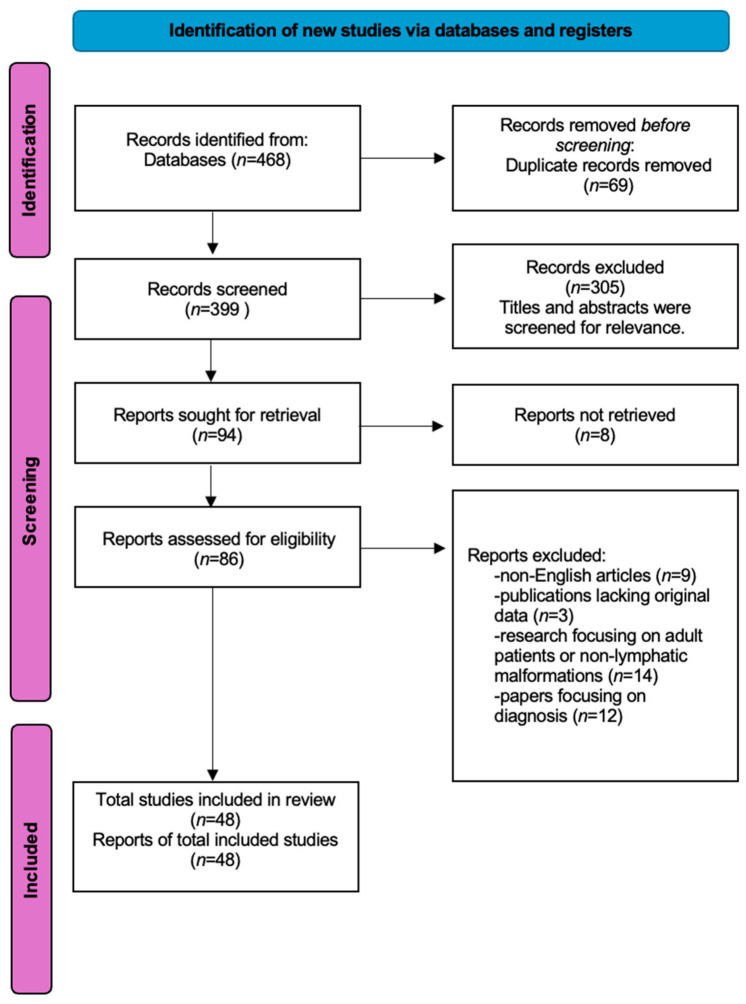
PRISMA flow diagram illustrating the study selection process.

In accordance with PRISMA guidelines, a comprehensive database search identified 468 records. After removal of duplicate records, titles and abstracts were screened for relevance. Full-text articles of potentially eligible studies were subsequently assessed according to predefined inclusion and exclusion criteria. Ultimately, 48 studies were included in the final review. The detailed study selection process is presented in the PRISMA flow diagram—Figure 3.

The identified therapeutic approaches were categorized into prenatal and postnatal interventions, each with its own advantages and limitations. Below, we provide a summary of the current evidence regarding management of this condition.

### 3.2. Prenatal Treatment

Most lymphatic malformations diagnosed prenatally are managed conservatively during pregnancy and treated postnatally. However, a subset of high-risk cases has stimulated interest in prenatal therapeutic interventions [16]. Current prenatal management strategies include intrauterine procedures aimed at decompression of the lesion, in utero sclerotherapy using agents such as OK-432, and pharmacologic interventions using mTOR inhibitors, including sirolimus (rapamycin). Despite encouraging outcomes in individual cases, prenatal therapy for lymphatic malformations remains experimental, with only limited case series available and no large-scale clinical trials to establish standardized protocols or assess long-term outcomes.

### 3.3. Prenatal Use of OK-432 (Picibanil)

Prenatal management of lymphatic malformations is still considered experimental but offers potential benefits in selected cases. The most commonly reported intrauterine therapy is the administration of *OK-432* (Picibanil), a lyophilized mixture of a low-virulence strain of *Streptococcus pyogenes*. OK-432 induces an inflammatory response within the cyst, leading to fibrosis and gradual lesion shrinkage. The therapeutic effect of this strain is believed to operate through cellular pathways and cytokine-mediated mechanisms [17].

We identified five studies reporting ultrasound-guided OK-432 injections performed between 21 and 32 weeks of gestation, frequently combined with the aspiration of cystic fluid. The outcomes of these studies are summarized in Table 2. Three of the analyzed studies reported a progressive reduction in tumor size [6,18,19]. In the study by Watari et al., no re-enlargement of the cysts was observed following OK-432 injection [20]. In the study by Chen et al., a neonate with recurrent fetal chylothorax who received two OK-432 injections developed cyanosis and respiratory distress immediately after birth and subsequently died, whereas a neonate with cystic hygroma treated with a single injection showed regression of the lesion and survived beyond four months of age at the time of manuscript submission [21]. Importantly, no maternal complications were reported in any of the included studies.

The effectiveness of OK-432 injections for the treatment of lymphatic malformations may depend on the neonate’s initial clinical status and the gestational age at diagnosis. According to Chen et al., a combination of antenatal OK-432 injection, maternal dietary modification, serial thoracocentesis, and paracentesis, along with amnioreduction and tocolysis, may enhance the success of antenatal therapy. Although OK-432 appears to be a promising therapeutic option, further research is required to assess its safety, effectiveness, and long-term effects on neonatal outcomes.

### 3.4. Prenatal Use of Sirolimus (Rapamycin)

Recent clinical experience suggests that sirolimus (rapamycin) may be a viable option for prenatal therapy in cases of severe vascular malformations. As an mTOR inhibitor with immunosuppressive properties, rapamycin suppresses the mTOR/phosphoinositide 3-kinase (PI3K) pathway, which plays a key role in regulating vasculogenesis [22]. Moreover, orally administered rapamycin crosses the placenta and acts directly on fetal tissues. Seront et al. reported comparable drug concentrations in both maternal and umbilical cord blood [26].

A report from the University Clinical Center in Gdańsk described two fetuses with extensive lymphatic malformations who were treated with sirolimus administered orally to the mothers during pregnancy. When given orally, the drug crosses the placenta and acts directly on the fetal tissues. The course of treatment was carefully monitored by measuring sirolimus (rapamycin) levels in both maternal and fetal blood. This maternal–fetal pharmacologic approach resulted in a significant reduction in the size and tension of the fetal lesions, allowing for safer delivery and improved postnatal outcomes. In both cases, postnatal surgery was avoided, and the infants continued sirolimus therapy after birth with favorable progression [27].

The effectiveness of this approach is further supported by additional case reports, demonstrating that maternal oral sirolimus therapy can reduce vascular malformations. Reports by Mégier et al. and Livingston et al. showed that prenatal rapamycin treatment led to a significant reduction in lesion size. Neither infant required postnatal surgery; one continued sirolimus therapy with favorable outcomes, while the other needed no postnatal treatment [22,23]. All studies included in this comparison are summarized in Table 2.

Across all reviewed studies, rapamycin effectively limited the growth of lymphatic malformations. The authors suggest that earlier initiation of sirolimus therapy may lead to even greater reductions in lesion size. Importantly, no adverse effects related to rapamycin use were reported. While these cases underscore the potential of sirolimus as a prenatal intervention, further research is required to establish its safety, efficacy, and standardized treatment protocols during pregnancy.

### 3.5. Prenatal Drainage and Decompression of Lymphatic Malformations

Other prenatal strategies for treating lymphatic malformations include serial aspiration, which involves repeated ultrasound-guided drainage of fluid from the lymphatic malformation over the course of gestation. This method is typically employed when the lesion is large, causing compression of adjacent structures, or when rapid growth is observed. The primary goal of this strategy is to reduce the size of the mass, prevent progression to hydrops fetalis, and avoid perinatal complications such as airway obstruction or dystocia [21,28].

In reported cases, aspiration was performed every 1–3 weeks, sometimes continuing until the onset of labor [24,25]. In both cases, the procedure successfully reduced cyst size and enabled safe delivery. Importantly, no adverse maternal or neonatal outcomes were reported in either study. All analyzed studies are summarized in Table 2.

Although serial aspiration does not address the underlying lymphatic malformation, it can effectively reduce lesion size and alleviate short-term symptoms. This approach may serve as a standalone palliative measure or as a temporary intervention prior to definitive postnatal treatment, such as surgical resection or sclerotherapy.

### 3.6. Postnatal Management

Postnatal management of lymphatic malformations represents the primary therapeutic approach for fetal lymphatic malformations and is determined based on lesion size, location, clinical symptoms, and genetic background. Available postnatal strategies include sclerotherapy; surgical excision; pharmacologic therapies; drainage; and, in rare cases, herbal or alternative approaches.

### 3.7. Sclerotherapy

Sclerotherapy is one of the most commonly applied postnatal treatments for lymphatic malformations. The agents most frequently utilized are bleomycin and OK-432, while doxycycline and ethanol appear only in select cases [3,7,28,29,30,31,32,33,34,35,36,37,38,39,40]. These agents act by inducing an inflammatory response and subsequent fibrosis within the cystic spaces, ultimately leading to volume reduction or complete regression of the malformation. A comparison of all commonly used sclerotherapy agents is presented in Table 3.

Bleomycin is a well-established sclerosing agent, commonly used in the postnatal treatment of large or septated lymphatic malformations. As a cytotoxic antibiotic, bleomycin targets the vascular endothelium, promoting fibrosis within lymphatic vessels and surrounding stromal tissue. While the exact mechanism underlying its sclerosing effect is not fully elucidated, it is thought that local inflammatory responses play a central role, which may occasionally lead to transient enlargement of treated lesions. Adverse effects are generally mild and transient, most commonly including flu-like symptoms, erythema, edema, and changes in skin pigmentation [17]. Bleomycin is particularly valuable in managing lesions that cause compression of adjacent structures, such as the airway, and sometimes combined with procedures such as EXIT (Ex Utero Intrapartum Treatment) to secure respiratory function immediately after birth [3,29]. The therapeutic response to bleomycin is typically gradual, with most lesions showing progressive regression over time. While generally well-tolerated, bleomycin may require repeated dosing and, in some cases, surgical intervention due to incomplete regression or recurrence [3,31].

OK-432 (Picibanil) is another commonly applied sclerosing agent in the treatment of lymphatic malformations, applied both prenatally and postnatally. In postnatal settings, OK-432 has been used either as monotherapy or in combination with other therapies such as doxycycline or surgical procedures [32,33]. The therapeutic effect of this agent is based on inducing a local inflammatory response, leading to fibrosis and shrinkage of the lesion. Compared with other sclerotherapeutic agents, OK-432 results in reduced fibrosis of the skin and subcutaneous layers. The adverse effect profile of this treatment is generally mild, typically limited to transient low-grade fever and local injection-site reactions, with no serious complications observed in neonatal or pediatric patients [6]. In the reviewed studies, OK-432 was often effective in reducing lesion size and, in some cases, led to complete resolution [35,36,37]. However, the effectiveness of this treatment appears to be variable, and a significant number of patients required subsequent surgical intervention despite initial sclerotherapy [33,37]. These results suggest that while OK-432 can be beneficial, it often serves as part of a multimodal treatment strategy rather than a definitive standalone solution.

Doxycycline is a less commonly used sclerosing agent in the treatment of lymphatic malformations. Although this agent belongs to the tetracycline class and is both accessible and cost-effective, the biological basis of its sclerosing action has not been fully clarified. In contrast to OK-432, treatment with doxycycline typically does not trigger a noticeable febrile or inflammatory response, indicating that it acts through alternative pathways. Current evidence suggests that doxycycline can inhibit matrix metalloproteinases and downregulate VEGF-driven angiogenesis and lymphangiogenesis, mechanisms believed to contribute to the progressive reduction in cystic lesions [17]. In one reported case, doxycycline was applied on its own and led to a satisfactory decrease in lesion size, with no need for further therapy [38]. In another instance, doxycycline was used during surgery in combination with OK-432, contributing to a positive outcome [32]. Although both cases showed encouraging short-term results, long-term follow-up data were not consistently available.

Ethanol is an easily accessible and potent sclerosing agent, but its clinical utility is limited by a high incidence of adverse effects. The strong cytotoxic and ischemic responses induced by ethanol can damage surrounding tissues, particularly cutaneous and neural structures, when alcohol diffuses beyond the targeted lesion. Compared with other sclerosants, ethanol is associated with markedly higher complication rates, including systemic toxicity. If absorbed into circulation, ethanol may provoke hypotension; symptoms of acute intoxication; or, in extreme cases, fatal outcomes. For this reason, ethanol’s use in young children with extensive malformations is generally avoided, as only minimal volumes can be administered safely—amounts that are often insufficient to achieve an adequate therapeutic effect [17]. Ethanol was mentioned in a single study describing its use in a case where complete surgical resection was not possible [40]. The outcome of the therapy was not fully detailed, and no additional data on safety or long-term effectiveness were provided.

### 3.8. Sirolimus

Three studies described the postnatal use of sirolimus, an mTOR inhibitor, in infants with extensive, often infiltrative, lymphatic malformations [32,41,42]. Sirolimus, commonly referred to as rapamycin, is a naturally derived macrolide originating from Streptomyces hygroscopicus that selectively inhibits the mammalian target of rapamycin (mTOR), a kinase critical for the regulation of cell proliferation and blood and lymphatic vessel formation. The antiproliferative and antiangiogenic characteristics of sirolimus provide a rationale for its use in the treatment of large lymphatic lesions [43]. In all cases, treatment resulted in a significant size reduction in the malformation, and none of the children required surgery [32,41,42]. However, the duration of follow-up was limited and, in some cases, final outcomes were not fully reported. Despite promising results, further studies are needed to evaluate the long-term efficacy and safety profile of sirolimus in this population. The overwiew of studies reporting postnatal sirolimus use is presented in Table 4. 

### 3.9. Surgical Intervention

Surgical treatment continues to play a key role in the management of lymphatic malformations, particularly in cases where the lesion causes airway obstruction, grows rapidly, or shows limited responses to conservative therapies such as sclerotherapy. Surgical resection was reported in numerous studies, with open surgery (laparotomy) being the most commonly used approach [2,38,44,45,46,47,48,49,50,51,52,53,54,55]. Laparoscopy was performed in only a small number of cases, suggesting it may be suitable for selected, less-extensive lesions [55,56]. Surgery was often performed after partial lesion reduction, following prior decompression or sclerotherapy [26,33]. In some cases, surgical excision was combined with adjunctive treatment, such as postoperative administration of bleomycin sclerotherapy at regular intervals following each resection stage [30,39]. The majority of patients undergoing surgery achieved favorable outcomes, although a few required additional procedures due to recurrence or residual lesions.

### 3.10. Laser Treatment

Laser excision may serve as an alternative option in the treatment of lymphatic malformations, particularly in anatomically challenging locations. The available data do not define the specific age group in which this method was applied. It was most commonly used for tongue lesions that were partially resected to reduce their volume. The reported recurrence rate following laser treatment was 40%, which was comparable to that observed after incomplete surgical excision [57]. While laser excision allowed partial control in select cases, it was not considered a curative approach and was associated with a relatively high risk of recurrence.

### 3.11. Drainage and Decompression

Postnatal drainage was described in a few studies as part of the therapeutic approach. In one case, drainage alone was sufficient and led to a satisfactory clinical outcome [58]. In other reports, drainage served as a supportive measure, typically combined with sclerotherapy using agents such as OK-432 or the herbal compound Eppikajutsuto [33,59]. In these cases, drainage was followed by surgical resection or further medical management, suggesting this treatment’s role as a temporary intervention rather than definitive treatment.

### 3.12. Other Medical Treatments

In rare cases, alternative or supportive treatments have been explored. One study described the use of Eppikajutsuto, a traditional Japanese herbal formula, administered after postnatal drainage of a mediastinal lymphatic malformation. While the lesion did not increase in size during follow-up, it also did not fully regress, suggesting a stabilizing but not curative effect [59].

### 3.13. Watchful Waiting and Spontaneous Regression

Although active intervention is often required in the management of lymphatic malformations, selected cases have demonstrated spontaneous regression without the need for surgical or pharmacologic treatment. This outcome has been observed in both recurrent lesions and neonates with cervical lymphatic malformations diagnosed early in life. In some clinical series, a watchful waiting approach was chosen postnatally, particularly when the malformations were not causing symptoms or functional impairment [57]. In most of these cases, the lesions gradually decreased in size and resolved within the first year of life [3,29]. These findings suggest that observation may be a reasonable option in selected, asymptomatic patients, especially during early infancy. However, the frequency of spontaneous regression appears to be low, and careful monitoring is essential to detect progression or complications.

## 4. Discussion

Effective treatment of fetal lymphatic malformations relies on an individualized, multidisciplinary approach. Although prenatal interventions such as OK-432 injections have shown encouraging outcomes in selected cases, they are still considered experimental and should be reserved for carefully selected candidates. Postnatal sclerotherapy remains the prevailing therapeutic approach, with agents such as bleomycin and OK-432 proving to be both effective and relatively safe, often reducing the need for surgical intervention. Systemic therapies, such as sirolimus, offer new possibilities for managing diffuse or inoperable lesions. However, further studies are necessary to confirm their long-term safety and efficacy. Ultimately, treatment decisions should be guided by lesion characteristics, the presence of associated anomalies or genetic findings, and the experience of the treating institution to ensure the best possible outcomes.

Furthermore, it is important to discuss the ethical dilemmas raised with the introduction of prenatal therapies. It is crucial to ensure that parents are adequately informed about the potential risks and benefits associated with the proposed interventions. For experimental treatments, it remains essential to obtain informed consent, which requires a solid scientific foundation and transparent communication with families. Additionally, these interventions should always be undertaken with respect for the patient’s autonomy and their right to make decisions. It is also important to consider impacts on the psychological well-being of mothers and families who may deal with uncertainty regarding treatment outcomes. Notably, the success of prenatal therapies is often context-dependent and does not always guarantee favorable results. Therefore, decisions regarding such interventions should be made based on interdisciplinary collaboration within the medical team.

Another important ethical consideration in the prenatal management of severe fetal lymphatic malformations is the legal framework governing pregnancy termination in the country where care is provided. In Poland, the current abortion law is highly restrictive. Termination of pregnancy is legally permitted only when the pregnancy poses a direct threat to the life or health of the pregnant woman or when there is a justified suspicion that the pregnancy resulted from a criminal act. Importantly, fetal anomalies, including severe or potentially lethal congenital malformations, do not constitute a legal indication for pregnancy termination under the current legislation. This legal context has significant ethical and clinical implications for the management of pregnancies complicated by extensive fetal lymphatic malformations. In cases where the prognosis is poor, therapeutic options are limited, and the risk of fetal or neonatal demise is substantial, parents and clinicians may face constrained decision-making pathways. 

The present study describes a rarely reported approach for the prenatal management of lymphatic malformations using intrauterine administration of bleomycin, a therapeutic agent conventionally reserved for postnatal sclerotherapy.

In the context of available prenatal therapeutic options, the choice of bleomycin in the present case should be interpreted in relation to alternative approaches reported in the literature. Intrauterine sclerotherapy using agents such as OK-432 has been described in a limited number of cases, with variable outcomes ranging from partial regression to perinatal death, reflecting both heterogeneity of lesions and the absence of standardized protocols. Similarly, prenatal pharmacologic therapy with sirolimus has shown promising results in isolated reports; however, its use remains experimental and is associated with limited data on dosing, timing, and long-term safety.

Other approaches, such as serial drainage or decompression, may provide temporary relief of mass effect but do not address the underlying pathophysiology of lymphatic malformations and often require repeated interventions. Surgical management, in turn, is not feasible in the prenatal setting and is reserved for postnatal care.

In this context, bleomycin was selected as a sclerosing agent with a well-established postnatal efficacy and safety profile, particularly in large and infiltrative lesions. Compared to other agents, its mechanism of inducing endothelial damage and fibrosis directly targets the cystic structure of lymphatic malformations, offering a potentially more definitive local effect than drainage alone. Although its prenatal use has not been previously reported, the decision to apply bleomycin in this case reflects a translational approach, adapting established postnatal therapy to a life-threatening fetal condition in the absence of validated prenatal alternatives.

Nevertheless, given the limited and heterogeneous evidence base, no single prenatal strategy can currently be considered superior. Therefore, the choice of intervention must remain individualized, taking into account lesion characteristics, disease progression, and the balance between potential benefits and risks.

In the present case, the intervention did not alter clinical outcomes, likely due to the extensive and multifocal nature of the malformation, the severity of fetal compromise, the advanced gestational age at time of treatment, and the limited interval between intervention and delivery. Nevertheless, prenatal sclerotherapy with bleomycin may represent a promising therapeutic option for carefully selected cases of severe fetal lymphatic malformations and warrants further investigation.

Importantly, the findings presented in this report should be interpreted with caution, as they are based on a single clinical case and therefore do not allow any conclusions regarding the efficacy, safety, or reproducibility of prenatal bleomycin therapy. The intrauterine use of bleomycin in fetal lymphatic malformations remains highly experimental and should currently be considered only in exceptional, carefully selected cases within experienced multidisciplinary centers. Further research, including larger case series and prospective studies, is necessary before this approach can be considered for broader clinical application.

## 5. Conclusions

While postnatal sclerotherapy of fetal lymphatic malformations continues to be the standard of care, emerging prenatal interventions may offer potential benefits in selected cases, although their safety and efficacy are not yet fully established.

The present case report introduces intrauterine bleomycin administration as a potential therapeutic approach in the management of fetal lymphatic malformations. This strategy expands the spectrum of potential prenatal interventions by adapting a well-established postnatal sclerosing agent to the intrauterine setting. However, based on the current experience, its clinical efficacy appears to be limited in advanced, extensive, or multifocal forms of lymphangiomas, particularly when significant fetal compromise is already present or when the intervention is performed late in gestation.

To provide more personalized and effective management of fetal lymphatic malformations, there is a strong need for earlier diagnoses, improved prognostic markers, and the development of standardized and effective treatment protocols, particularly for prenatal and emerging therapies. Equally important is a multidisciplinary approach to parental counseling and clinical decision-making. Together, these elements should form the foundation of comprehensive clinical management in such complex cases.

Given that the present report reflects only a single clinical experience, no generalized conclusions regarding therapeutic effectiveness can be drawn. Further experimental and clinical studies are required to evaluate the safety, feasibility, and potential role of prenatal bleomycin therapy in fetal lymphatic malformations.

## Figures and Tables

**Table 1:** Diagnostic criteria of mirror syndrome according to the systematic review by Biswas et al. [15].

Criteria	Number of Studies That Included This Criterion in the Diagnosis of Mirror Syndrome	Definition
Maternal edema	11/12	Weight gain of more than 1 kg/wk Accumulation of excessive fluid within subcutaneous and body cavities, weight gain, and preeclampsia-like symptoms Skin and/or pulmonary edema Based on clinical examination
Fetal hydrops	9/12	Pathologic fluid accumulation in ≥2 different fetal compartments
Placental edema	6/12	Placenta that was 90% heavier than the normal range Confirmed by pathology
Placentomegaly	5/12	Ultrasound-detected placental thickness of ≥40 or ≥60 mm in the second and third trimesters of pregnancy, respectively Placenta thicker than 4−5 cm over its entire extent More than 6 cm thickness after 21 wk of gestation More than 5 cm thickness More than 6 cm thickness
Preclampsia	2/12	Edema, elevated blood pressures, proteinuria, abnormal liver function tests, and abnormal platelet count

**Table 2:** Prenatal treatment of lymphatic malformations.

OK-432 Sclerotherapy					
Author	Type of Study	Study Group	Intervention	Results	Complications
Mikovic et al., 2009 [6]	Case Report	Two patients with large multicystic neck lymphangiomas.	Single intralesional injection of OK-432.	A progressive decrease in tumor volume throughout gestation.	No maternal or fetal complications were noted.
Ogita et al., 2001 [18]	Case Series	Two patients with fetal cystic hygromas.	OK-432 injection under ultrasound guidance.	The size of the cyst decreased in 1 of 2 cases treated in utero.	There was no information about fetal or maternal complications.
Chen et al., 2005 [21]	Case Series	Patient 1: recurrent fetal chylothorax. Patient 2: cystic hygroma.	Patient 1: OK-432 injection into the fetal pleural cavity at GA 33 and 34 weeks. Patient 2: OK-432 injection into the tumor at GA 23 weeks.	Patient 1: The baby expired despite aggressive neonatal resuscitation. Patient 2: regression of cystic hygroma. The baby had survived beyond 4 months of age at submission.	Patient 1: cyanosis and respiratory distress immediately after birth. Patient 2: fetal ascites and chylothorax, which resolved within two days after birth. No adverse maternal outcomes were reported.
Watari et al., 1996 [20]	Case Report	One patient with two cystic hygromas.	Removal of intracystic fluid from both cysts and injection of OK-432 at 21 and 28 weeks of gestation.	No re-enlargement of the cysts was subsequently observed.	Only a slight skin fold was observed in the nuchal area of the neonate. No adverse maternal outcomes were reported.
Sasaki et al., 2003 [19]	Case Report	One fetus with large multiple cysts around the neck.	Injection of OK-432 at 29 and 32 weeks of gestation.	The cysts decreased in size after intrauterine treatment.	At birth, the neonate presented with minor nuchal skin swelling, which had completely resolved by one month of age. No adverse maternal outcomes were reported.
Sirolimus (Rapamycin)					
Mégier et al., 2025 [22]	Case Report	One fetus with large fetal axillary cystic lymphatic malformation.	Maternal oral rapamycin administration for 10 days.	The cysts decreased in size after the intrauterine treatment.	Wrinkled skin at the location of the lymphatic malformation; a small mass of approximately 1 cm was palpable on the left axilla of the neonate. No adverse maternal outcomes were reported.
Livingston et al., 2021 [23]	Case Report	One fetus with a large cervical lymphatic malformation detected during a routine 20 week anatomy ultrasound.	Maternal oral rapamycin was administered for 39 days, followed by continued therapy for the neonate after delivery.	Cessation of mass growth and stabilization of polyhydramnios	There were no significant maternal, fetal, or neonatal side effects from rapamycin.
Drainage and Decompression of the Lymphatic Malformation					
Kaufman et al., 1996 [24]	Case Report	One fetus with a large cystic chest wall mass.	The cystic mass was aspirated under ultrasound guidance.	The cystic mass was reduced in size, and the patient underwent an uneventful induction of labor.	No maternal or fetal complications were reported.
Chen et al., 1996 [25]	Case Series	Two patients with large, persistent, isolated, fetal nuchal cystic hygromas.	The patients underwent echo-guided lymphatic drainage by fine-needle aspiration during the second and third trimesters.	The cystic masses were successfully reduced in size.	No maternal or fetal complications were reported.

**Table 3:** Comparison of agents used in postnatal sclerotherapy.

Agent	Mechanism of Action	Effectiveness	Adverse Effects
Bleomycin	Induction of fibrosis within lymphatic vessels and the surrounding stromal tissue [17].	Bleomycin typically induces gradual lesion regression, often requiring repeated doses, and surgery may be needed to resolve incomplete responses or recurrence [3,31].	Flu-like symptoms, erythema, edema, and changes in skin pigmentation [17].
OK-432 (Picibanil)	Triggers a localized inflammatory response that results in fibrosis and subsequent lesion shrinkage [17].	Effectiveness is variable, and many patients still require surgery after initial sclerotherapy [35,36,37].	Transient low-grade fever and localized injection-site reactions have been reported; no serious complications have been observed in neonatal or pediatric patients [6,17].
Doxycycline	Its sclerosing effect is not fully understood but may involve inhibition of matrix metalloproteinases and downregulation of VEGF-driven angiogenesis and lymphangiogenesis, contributing to cyst regression [17].	Variable efficacy reported in studies [32,38].	Pain on injection, erythema, edema, and dental staining [17].
Ethanol	Induces strong cytotoxic and ischemic responses [17].	In one case report mentioning the use of methanol, the therapeutic outcomes were not fully described [40].	Nerve injuries, ischemia, skin necrosis, and systemic effects such as hypotension, respiratory depression, cardiac arrhythmia, and hypoglycemia [17].

**Table 4:** Overview of studies reporting postnatal sirolimus use.

Author	Type of Study	Study Group	Intervention	Results	Complications
Garcia-Diaz et al., 2020 [32]	Retrospective review	Patient 1: a newborn with a large cervical lymphangioma. Patient 2: a newborn with a large bilateral cervical cystic lymphangioma with polyhydramnios.	Patient 1: treatment with sirolimus (0.04 mg/m^2^/day) on the 10th day of life. Patient 2: Treatment with sirolimus was started from the 8th day of life.	Patient 1: There was no need for surgical resection, and the progress of the child was good, according to the authors. Patient 2: There was no need for surgical resection, and the progress of the child was good according to the authors	Patient 1: no adverse maternal and neonatal outcomes were reported. Patient 2: no adverse maternal and neonatal outcomes were reported.
Amodeo et al., 2017 [42]	Case Series	Patient 1: a newborn with lymphatic malformation extending from the posterior area of the neck to the lower abdomen. Patient 2: a newborn with giant multicystic lymphangioma displacing the trachea and the larynx. Patient 3: a newborn with lymphangioma in the left cervical and upper mediastinal region and accompanying hydrothorax. Patient 4: a newborn with multicystic lymphatic malformation in the left cervical region and upper portion of the left anterior thorax.	Patient 1: At 3 months of age, pharmacological therapy with sirolimus was started. Patient 2: At 16 months of age pharmacological therapy with sirolimus was started. The dosage was 0.4 to 0.8 mg/m^2^, 2 times/day. Patient 3: At 23 months of age pharmacological therapy with sirolimus was started. The dosage was 0.4 to 0.8 mg/m^2^, 2 times/day. Patient 4: Treatment with sirolimus was started on the 13th day of life and later continued after surgery.	Patient 1: reduction in mass size after 3 months of treatment. Patient 2: Despite gradual dose escalation, therapeutic blood levels were difficult to maintain. The patient remains under ongoing clinical, pharmacological, and radiological follow-up. Patient 3: after 6 months of treatment, an overall reduction in the cysts and the spleen diameter was appreciated. Patient 4: After 2 months of treatment, the MRI scan with contrast documented an overall involution to microcystic lesions	Patient 1: Moderate dyslipidemia with elevated cholesterol and triglycerides developed after six months of treatment. Patient 2: No adverse maternal and neonatal outcomes have been reported thus far. Patient 3: After 6 months of treatment with sirolimus, the patient developed mild dyslipidemia with high levels of cholesterol and triglycerides. Patient 4: As an isolated adverse effect of sirolimus treatment, the patient developed mild dyslipidemia with high levels of cholesterol.

## Data Availability

The data that support the findings of this study are available from the corresponding author upon reasonable request.
